# Supplementary figures and images for: Gut Microbial Diversity Reveals Differences in Pathogenicity between Metarhizium rileyi and Beauveria bassiana during the Early Stage of Infection in Spodoptera litura Larvae
Source: Microorganisms. 2024 May 31;12(6):1129. doi: 10.3390/microorganisms12061129 (PMC11206097; doi:10.3390/microorganisms12061129)

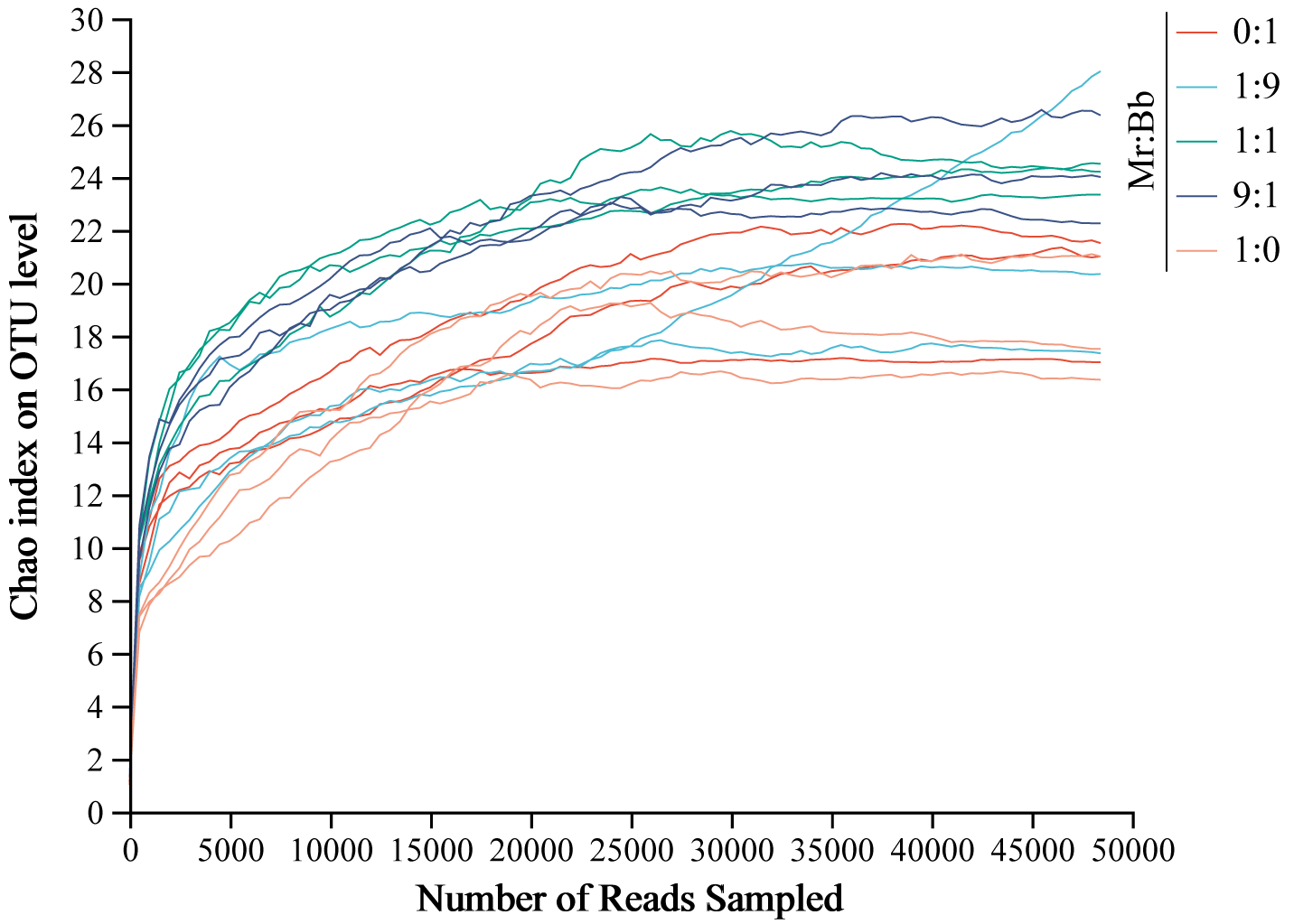

Supplement: Supplementary file 1 [file microorganisms-12-01129-s001.zip › Figure S1.tif]

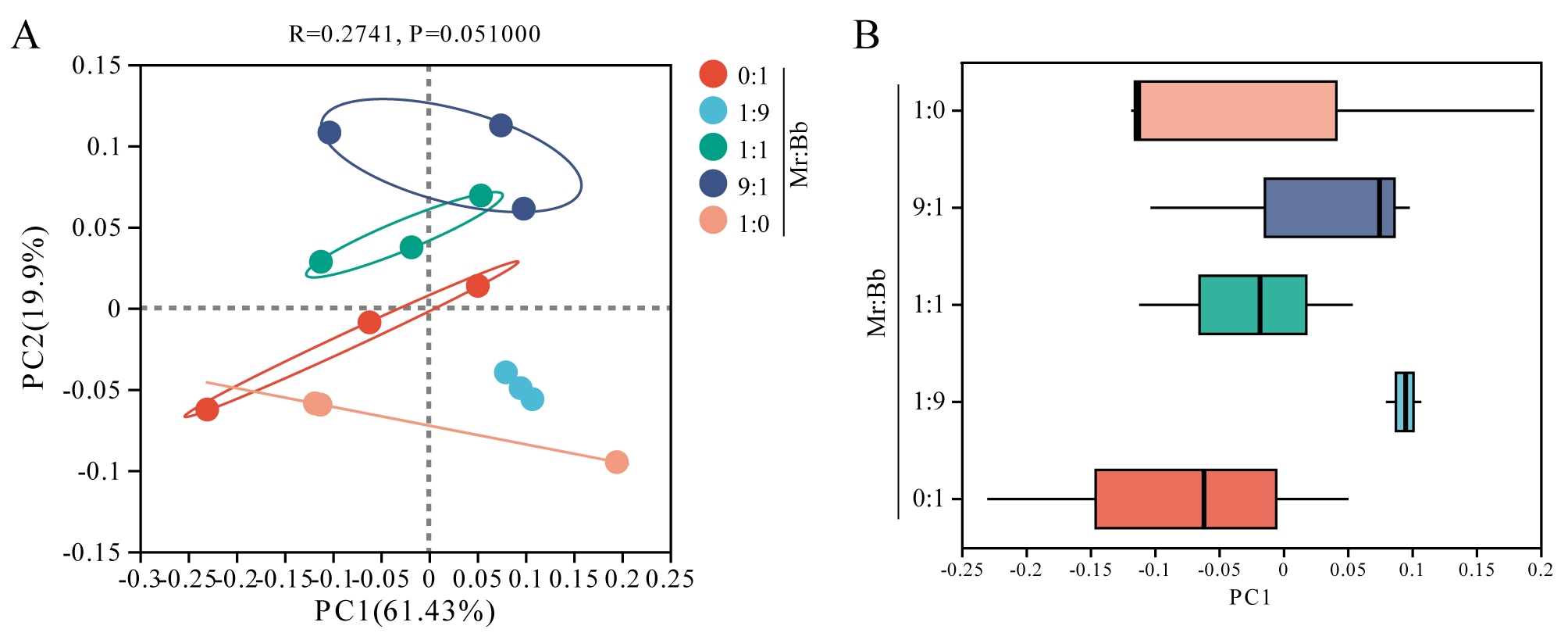

Supplement: Supplementary file 1 [file microorganisms-12-01129-s001.zip › Figure S2.tif]
